# Biased Ghrelin Receptor Signaling and the Dopaminergic System as Potential Targets for Metabolic and Psychological Symptoms of Anorexia Nervosa

**DOI:** 10.3389/fendo.2021.734547

**Published:** 2021-09-27

**Authors:** Mariam S. Khelifa, Louise J. Skov, Birgitte Holst

**Affiliations:** Department of Biomedical Sciences, University of Copenhagen, Copenhagen, Denmark

**Keywords:** ghrelin, dopamine, anorexia nervosa, biased signaling, food intake, gut motility, anxiety, locomotion

## Abstract

Anorexia Nervosa (AN) is a complex disease that impairs the metabolic, mental and physiological health of affected individuals in a severe and sometimes lethal way. Many of the common symptoms in AN patients, such as reduced food intake, anxiety, impaired gut motility or overexercising are connected to both the orexigenic gut hormone ghrelin and the dopaminergic system. Targeting the ghrelin receptor (GhrR) to treat AN seems a promising possibility in current research. However, GhrR signaling is highly complex. First, the GhrR can activate four known intracellular pathways Gαq, Gαi/o, Gα12/13 and the recruitment of β-arrestin. Biased signaling provides the possibility to activate or inhibit only one or a subset of the intracellular pathways of a pleiotropic receptor. This allows specific targeting of physiological functions without adverse effects. Currently little is known on how biased signaling could specifically modulate GhrR effects. Second, GhrR signaling has been shown to be interconnected with the dopaminergic system, particularly in the context of AN symptoms. This review highlights that a biased agonist for the GhrR may be a promising target for the treatment of AN, however extensive and systematic translational studies are still needed and the connection to the dopaminergic system has to be taken into account.

## 1 Introduction

Anorexia nervosa (AN) is a complicated disease that has both psychological and metabolic components. AN is the psychiatric disease with the highest mortality rate, however no good drug treatment is currently available (1–3). AN is associated with a cluster of symptoms including metabolic dysfunctions, such as decreased appetite, underweight, a high level of physical activity, and impaired gut motility in addition to mental alteration such as increased anxiety (4–6). The GhrR signaling in the central nervous system is closely related to the DAergic mesocorticolimbic system, which itself is often discussed in the context of AN (3, 7). The connection of AN symptoms to ghrelin and the dopaminergic (DAergic) system has been drawn before (4, 8–11). However, how to exploit this connection in order to target the disease with for instance a ghrelin receptor (GhrR) agonist is still not well studied and has led to unsatisfying/inconclusive results so far (3). The GhrR can activate multiple different signal pathways, which allow for targeting biased signaling properties, by only activating a subset of the available signaling pathways. With biased signaling it may be possible to specifically target the core symptoms without adverse effects [the potential of biased signaling was reviewed (12)]. Further, it may be possible to not only target primary symptoms by increasing the motivation to eat and food intake, but also target secondary symptoms, such as impaired gut motility, anxiety and overexercising, which may lead to increased subjective well-being of the treated individuals. However, in order to target the central symptoms of AN with a biased GhrR agonist, it is necessary to understand the physiological function of all the GhrR signaling pathways in the context of AN.

## 2 Energy Balance

Maintaining energy balance is crucial for all living organisms. The equilibrium between energy expenditure (e.g., physical activity or maintaining adequate body temperature) and energy intake needs to be highly regulated. Disturbances in the energy balance can result in overweight caused by too high energy intake or too little expenditure and underweight due to too low energy intake or too much expenditure. Both imbalance types affect the physical health drastically and can lead to systemic organ failure (1, 13). Disturbances of the balance towards too low energy intake can be due to various underlying pathophysiological causes, such as cancer-induced cachexia or seemingly self-induced as is the case for AN (6, 14). Insufficient energy intake over a longer period of time will result in the malfunctioning of cellular processes, which is then reflected in the impairment of basic physiological functions, such as muscle capacity or fertility and may ultimately result in death (15). This underlines why the energy balance is one of the most complex regulated systems in the body, particularly in ensuring a sufficiently high energy intake/storage. The regulation of appetite is considered to be orchestrated by two different systems: the homeostatic and the hedonic system.

### 2.1 Food Intake Regulated by the Homeostatic and Hedonic System

#### 2.1.1 Homeostatic Regulation of Food Intake

**Figure 1:**
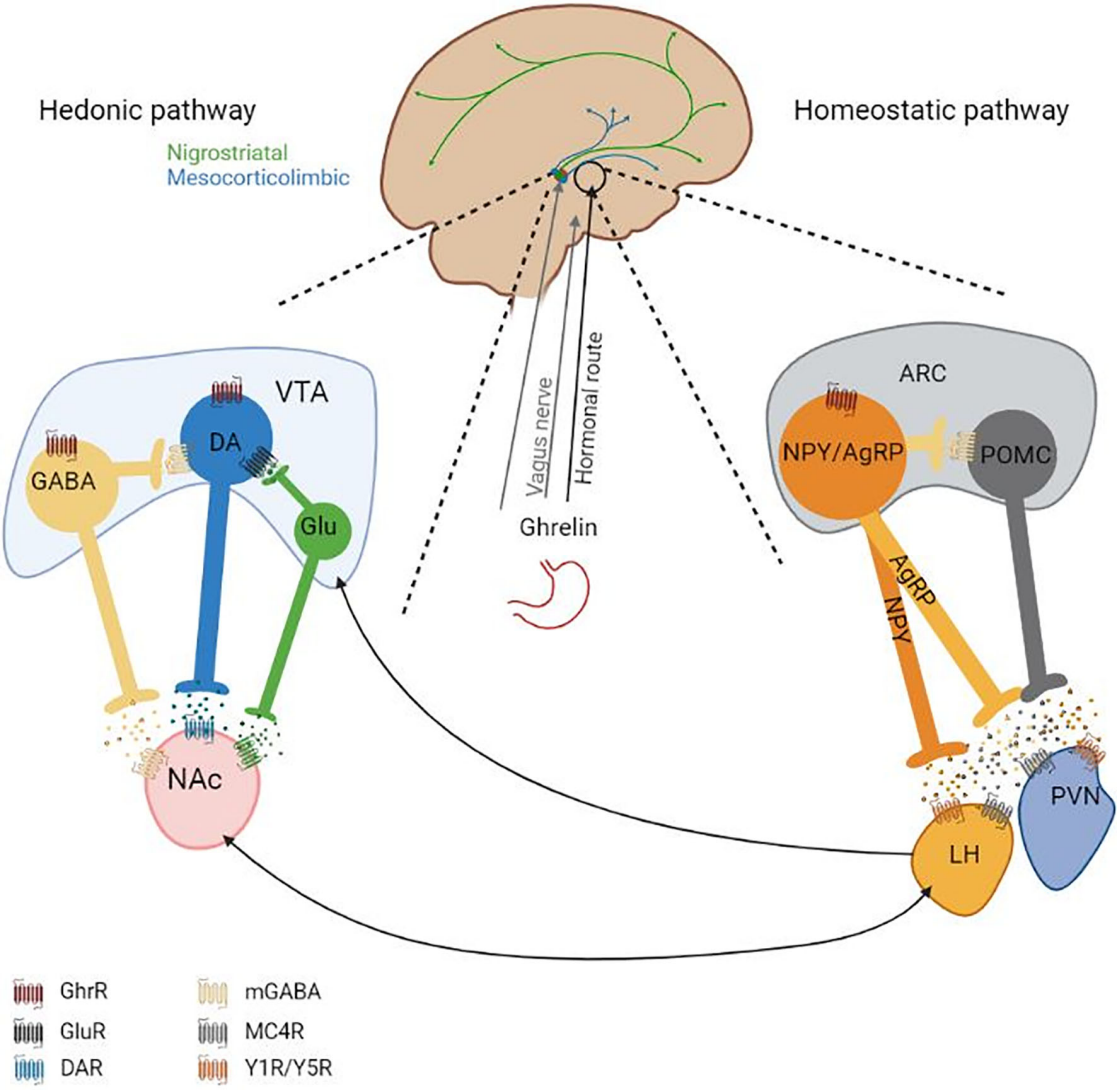
Overview of (potentially) ghrelin-modulated appetite-regulating pathways. Ghrelin reaches the homeostatic pathway (on the right) *via* the peripheral circulation (hormonal route) and potentially vagus nerve. How and if ghrelin signaling reaches the VTA remains unknown. *On the left:* It is generally considered that ghrelin leads to the induction of DA release from the VTA to the NAc in the presence of food, but there are inconclusive findings under the absence of food. The VTA has further neuronal projections to other brain regions. The DAergic system, including anxiety or locomotion modulating circuits, is highly interconnected. *On the right:* In the ARC the two depicted neuronal populations have opposing effects regarding the regulation of food intake. Activation of NPY/AgRP neurons induces food intake and activation of POMC neurons leads to the suppression of food intake. Ghrelin binds GhrRs on NPY/AgRP neurons in the ARC and activates them which leads to the release of GABA, NPY and AgRP. GABA inhibits POMC neuronal activity, which leads to the reduction of α-MSH release. α-MSH induces neurotransmitter release in the second-order neurons located in e.g. the LH and PVN *via* binding to MC4R. AgRP is a MC4R antagonist, reducing neurotransmitter release from the second-order neurons. NPY binds Y1R or Y5R on second-order neurons, which inhibits neurotransmitter release. Second-order neurotransmitters affect other central regions, which will further mediate the orexigenic effect of ghrelin or anorexigenic effect of other factors (e.g. leptin). The interconnectivity of the hedonic system and the homeostatic system is indicated with arrows. AgRP, Agouti releasing peptide; ARC, arcuate nucleus; DA, Dopamine; GABA, γ-Aminobutyric acid; GhrR, Ghrelin receptor; LH, Lateral hypothalamus; MC4R, Melanocortin-4-receptor; mGABAR, Metabotropic GABA receptor; NAc, Nucleus accumbens; NPY, Neuropeptide Y; POMC, Proopiomelanocortin; PVN, Paraventricular nucleus; VTA, Ventral tegmental area; Y1R/Y5R, Neuropeptide Y receptor Y1 and Y5; α‐MSH, α-Melanocyte-stimulating hormone. Figure was created with BioRender.com.

To maintain the energy balance, the homeostatic system is responsible for the main regulation of food intake. A major regulation area of the homeostatic food intake is the hypothalamus, where different hunger and satiety signals influence appetite and thus the food intake. The incoming signals originate from different central and peripheral locations and include factors such as blood glucose or gastrointestinal content, which are indicators for the body’s energy state. Between the periphery and the hypothalamus, a communication is mediated through hormonal signaling and the vagus nerve. The vagus nerve has projections from a variety of peripheral locations, e.g. from the digestion tract, to the nucleus tractus solitarius (NTS) in the brain stem from which further projections reach different brain regions including the arcuate nucleus (ARC) and the lateral hypothalamic area (LH) in the hypothalamus. ARC constitute a central hypothalamic site for the regulation of appetite. Its proximity to the median eminence allows the ARC to receive hormone signaling, as the blood-brain barrier is leaky and is penetrable for peripheral peptides. In the ARC, two first-order neuronal populations are best described in their function as appetite regulators, first, propiomelanocortin (POMC) neurons, expressing α-MSH, which leads to the suppression of food intake when activated. Second, neurons that co-express neuropeptide Y (NPY) and agouti-related peptide (AgRP) that increase food intake when activated. Both neuronal populations have projections to neurons located in the second-order areas e.g. the paraventricular nucleus (PVN) or the LH, however they have opposing effects on the neurons in these areas (**Figure 1**). Being both connected with the periphery and other central areas the ARC is an important link between peripheral signaling to central signaling. The communication from the periphery is due to various hormones and neuroendocrine peptides such as leptin, GLP-1, neurotensin (all anorexigenic) or ghrelin (orexigenic) to name a few (15–17).

#### 2.1.2 Hedonic Regulation of Food Intake

The hedonic food intake regulation is dependent on the mesocorticolimbic system which originates in the VTA of the midbrain and is part of the DAergic system. From the VTA, the DAergic neurons project to the nucleus accumbens (NAc) of the striatum. The DAergic system is pivotal for motivational/food seeking behavior. Mice with a dopamine (DA) deficiency die when they are three weeks old due to lack of food seeking behavior regardless of functional locomotion and food ingestion capability (18). The “dopamine hypothesis” states that DA signaling helps to focus the attention on environmental stimuli and leads to a motivational drive to obtain (or avoid these stimuli). Hence, increased DA signaling will lead to a higher motivational drive. This drive can even overrule other internal signals, such as the homeostatic regulation of appetite (19). In the VTA there is a comparably high expression of receptor for peripheral hormones on both DAergic neurons and GABAergic neurons and intra-VTA administrations of these peripheral hormones lead to an alteration of food intake (20–22). This could imply that the VTA is under peripheral modulation. However, some of these hormones are also centrally produced and therefore act as neurotransmitters, e.g. GLP‐1 and neurotensin containing neurons in the NTS and LH, respectively (23, 24). Additionally, other hormones, such as ghrelin are not able to pass the blood-brain barrier, it was demonstrated that a peripheral administration of ghrelin does not increase the cFos expression in the VTA (25). It has been argued that ghrelin is centrally produced, however, these results are still controversial as other studies have not been able to confirm but rather contradict these findings (26, 27). Generally, it has been suggested that ghrelin has an excitatory effect on DAergic neurons in the VTA, which leads to DA release in the NAc in the presence of food and blockage of the GhrR signaling has been shown to inhibit this DA release (28, 29) (**Figure 1**). Potentially, other pathways of the DAergic system are influenced by GhrR signaling, however with the current knowledge this is rather a suggestion (7).

#### 2.1.3 Connection Between the Homeostatic and Hedonic Systems

Both the homeostatic system and the hedonic system are crucial to maintain a balanced food intake. However, describing the homeostatic and hedonic appetite regulation systems as two seemingly separate systems is a simplification. The second-order neurons located in the PVN and the LH are known to have projections to the VTA and the NAc (30). The two systems have been shown to communicate and influence each other. For instance, an ablation of AgRP circuitry alters DA signaling and DA-associated behaviors (31). Generally, the DAergic appetite-regulating system has been suggested to overpower homeostatic signals, in for example reward-related and palatable food intake (19). Furthermore, a disease like AN indicates that regardless of hunger signals, the patients are able to barely eat and even starve themselves to death. That a dysregulated DAergic system may play a role in the altered regulation of food intake in AN patients has been suggested by some studies (9, 11). GhrR signaling has been demonstrated to be a mediator in both the hedonic and the homeostatic regulation of food intake (**Figure 1**).

## 3 Anorexia Nervosa

It is established that Anorexia Nervosa (AN) is a multifactorial disease with genetical and environmental components. The development of AN is argued to be a complicated interwoven net of sociocultural, psychiatric, and in the recent years discussed more prominently: metabolic factors (4, 32). A genome wide association study has further solidified AN as a metabo-psychiatric disorder (33). Being such an interdisciplinary and multilevel disease could explain why the etiology of AN is still poorly understood. However, both the DAergic and the ghrelin systems are promising in their potential to elucidate underlying mechanisms and the etiology of AN. Individuals suffering from AN are characterized by three core symptoms: restricted food intake leading to underweight, a disturbed body image, i.e., feeling overweight despite being underweight and an intense, irrational anxiety of gaining weight (3, 6, 15, 32). The drastic insufficiency of energy intake can result in damages affecting every organ system, which is responsible for the fact that AN is the psychiatric disease with the highest mortality rate (1, 2). Nevertheless, treating AN with medical intervention has been proven difficult and with little promising outcome, often due to a lack of compliance by the patients (3, 34).

AN can be divided into two subtypes, the restricted type, and the binge/purge type. The restricted type shows a restricted eating behavior typically combined with hyperactivity (overexercising). Binge/purge AN patients display binge eating followed by actions that interfere with energy intake e.g. vomiting. Around 40-45% of the binge/purge AN patients can be classified as compulsive exercisers, for the restricting type it is 80% (6, 35). As locomotion is closely related to ghrelin and DA signaling, this review mainly focusses on the restricting AN type. An animal model which reflects the restricted type quite well is the activity-based anorexia (ABA) animal model in which rodents have both restricted feeding and unlimited access to a running wheel. This will lead to the development of AN-like behavior with the rodents losing a significant amount of weight and expressing hyperactivity which will ultimately result in their death due to a severely impaired energy Balance (36).

## 4 The Dopaminergic System

### 4.1 The Physiological Functions of Dopamine

The DAergic system in the brain consists of four different pathways, the mesolimbic pathway, the nigrostriatal pathway, the mesocortical pathway and the tuberoinfundibular pathway. In the context of AN, the motivational pathway (mesocorticolimbic; combination of mesolimbic and mesocortical pathway) is most relevant, due to its regulatory effect on motivation or “wanting” of food (37, 38). Additionally, the nigrostriatal pathway must be involved too, as food seeking behavior is not only the motivation to obtain food, but also the locomotive behavior to obtain it. Furthermore, DAergic signaling, as ghrelin signaling, generally has modulatory effects on anxiety and gut motility (39–41). The DAergic neuronal circuits are complex systems which show a high interconnective capacity (30, 42). For these neuronal circuits DA is the main neurotransmitter which can activate different signal pathways depending on the activated neuronal sub-populations or the receptor type DA binds to.

### 4.2 D1R *vs* D2R – The Counteractive Actions of the Two Dopamine Receptor Classes

DA binds to and acts *via* two different receptor classes, the D1 receptor class (D1R and D5R) and the D2 receptor class (D2R, D3R and D4R). Both receptor classes are G protein-coupled receptors (GPCRs) but they activate opposite intracellular signaling pathways. D1R is Gαs-coupled, and its activation will lead to adenylate cyclase activation which results in the accumulation of 3′,5′-cyclic adenosine monophosphate (cAMP) and activation of the neuron it is expressed on. D2R is Gαi/o coupled, which leads to a decrease in cAMP and inhibition of the neuron (43). Both D1R and D2R can recruit β-Arestin’s (43). D2R is expressed in multiple brain areas and can be expressed both pre- and post-synaptic. The presynaptic expression of D2R induces a negative feedback loop and its postsynaptic expression inhibits neurotransmitter release from both DAergic and non-DAergic neurons (44). D1R is only expressed post-synaptic. D1R and D2R are differently expressed throughout brain regions and neurons but can be co-expressed. In the VTA, both D2R and D1R are expressed. However, D1R is most highly expressed in the dorsal and ventral striatum. D2R is expressed on presynaptic neurons in the VTA and on postsynaptic neurons, e.g. on a subset of medium spiny neurons in the NAc (part of the ventral striatum), which are targeted by DAergic projections from the VTA (44).

### 4.3 Altered Dopaminergic System in Anorexia Nervosa Patients

Individuals affected by AN display a behavior that appears to be closely related to dysregulation of the mesolimbic system and motivational control. AN patients generally show a high rate of self-control and are less sensitive to rewards and more sensitive to punishment (9, 45). However, other studies have found increased brain activity of the DA-mediated reward system in AN patients compared to healthy controls when exposing the subjects to situations that require reward-related learning (46). A recent study has observed that the altered reward response is inversely associated with the body mass index in eating disorder patients, including AN patients. Further, they report changed signaling of reward and food intake brain circuits in AN patients compared to healthy controls (47). Overall, indications of altered DA signaling in AN patients are given. Nevertheless, it is difficult to determine whether the DA signaling is generally increased or decreased. For instance, in the previously described study with DA deficient mice, the mice showed both hypoactivity and hypophagic behavior before dying when three weeks old due to lack of food seeking behavior (18). These findings are both parallel and antiparallel to typical AN behavior as the patients show lack of food seeking behavior, however AN is associated with increased activity levels (6, 35, 36). A recent fMRI study in women demonstrated that the VTA activity, measured with the blood flow to the VTA, in AN patients is reduced compared to bulimic patients when exposed to food odors, which may indicate the potential dysregulation of VTA activity in AN patients (9). Additionally, a retrograde viral injected, chemogenetic induction the Gαq activity in projections from the VTA to NAc led to increased food intake and food anticipatory behavior in ABA rats. This neuron activation was mainly due to activated DAergic neurons (11). These results imply that a decreased activation of the DAergic projections from VTA to the NAc, which are known to be activated by ghrelin in the presence of food, may be part of the AN etiology and an activation/modulation of these projections may reduce AN development (28). However, it was observed that a knockout of the DA transporter, leading to an increase in extracellular DA, led to a stronger progression of AN-associated phenotypes in female ABA mice. This indicates that an increase in DA release might be relevant for the initial development of AN, which suggests a dual role of DA in the etiology of AN (48). Due to the promising link between AN and the DAergic system, various studies have looked into the potential of antipsychotic drugs (D2R receptor antagonists). However, no overall positive effect was observed in a recent meta-analysis (3).

## 5 Ghrelin

Ghrelin is an orexigenic gut hormone that was first identified in the stomachs of rats over 20 years ago (49). Since then, it has been shown that ghrelin mRNA is widely expressed in multiple tissues and its main production site is X/A-like cells in the fundus of the gastric lumen (50, 51). Ghrelin is a 28 amino acid long peptide modified by an eight or ten carbon fatty acid chain, which is crucial for the binding and activation of its receptor. The posttranslational fatty acid modification is catalyzed by an enzyme called ghrelin O-acyltransferase (GOAT) (52, 53). Ghrelin can modulate a variety of physiological functions by activating the GhrR which is expressed in multiple different tissue types and organs.

### 5.1 The Physiological Function of Ghrelin

Ghrelin is most prominently studied in regard to its growth hormone-releasing, food intake- and energy balance-regulating functions. However, ghrelin is a hormone with various physiological and behavioral modulating effects. Most interestingly, some of the physiological functions of ghrelin are highly connected to characteristic AN symptoms. Ghrelin is known to increase food intake by modulating the homeostatic and hedonic systems and it is also known to increase gut motility. Furthermore, ghrelin is involved in stress and reward-oriented behaviors and regulates anxiety and locomotion (54, 55). Since ghrelin specifically increases the pleasure and motivation related to food intake, which is relevant for AN patients, it is possible that an agonist of the GhrR could be a potential pharmacological target. Studies examining the effect of GhrR targeting in cancer induced cachexia have shown promising results regarding increasing body weight (BW) and muscle strength (56, 57). However, clinical trials with AN patients have demonstrated that the hunger-inducing effect of the full GhrR agonists in this sensitive population of patients may not be tolerated unless it is accompanied by other beneficial effects, such as alleviation of anxiety and gastro-intestinal dysfunction (34). Accordingly, more research must be done to find out how to target the GhrR pathways in order to enhance gut motility, lower anxiety and keep locomotion at least neutral in AN patients while still enhancing food intake in a healthy way that is tolerable to the patients. However, many of the studies examining the exact physiological and behavioral functions of ghrelin still show inconsistent results.

### 5.2 The Complex Signaling of the Ghrelin Receptor

**Figure 2:**
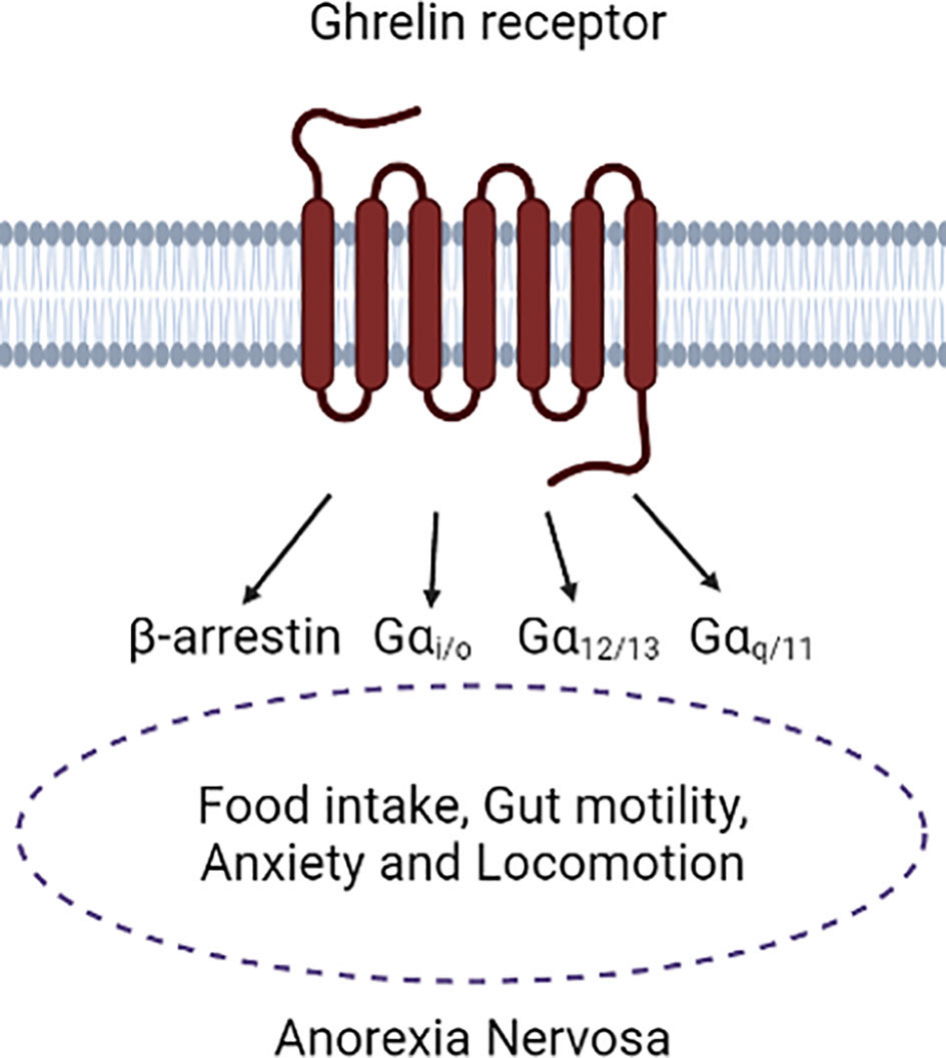
Depiction of the ghrelin receptor in association with Anorexia Nervosa symptoms. It is mainly known that a connection of ghrelin receptor signaling to Anorexia Nervosa symptoms is present, however the complete translation of the different intracellular pathways to the physiological functions is still needed. Figure was created with BioRender.com.

The GhrR is expressed in different tissues both peripherally, e.g. in the pituitary gland or pancreatic islets and centrally, most importantly for AN in brain regions related to food intake and anxiety behavior, such as the hypothalamus, the VTA and the amygdala (50, 58–60). The GhrR is a member of the Class A Rhodopsin-like GPCRs and the signaling of the GhrR is very complex (61, 62). The first discovered and best studied pathway is the increased Ca^2+^ mobilization after activation of the GhrR when inducing growth hormone release in the pituitary gland (63). This effect was traced back to the activation of the Gαq/11-coupled pathway, which initiates the activation of phospholipase C, which will then activate the inositol 1,4,5-trisphosphate and diacylglycerol pathways, which both ultimately result in increased Ca^2+^ mobilization, which increases neurotransmitter release in neurons (64, 65). This discovery was followed by further observation demonstrating that GhrR signaling pathways additionally include Gαi/o, Gα12/13 and the recruitment of β-arrestin (**Figure 2**), where Gαi/o-coupling may initiate neuronal inhibition. Signaling of Gα12/13 is often considered as one category even though they have different signaling properties (66, 67). It has been shown that Gα12/13 activity is often involved in cytoskeletal rearrangement (68). β-arrestin recruitment is part of a negative feedback mechanism and is not mediated *via* the G proteins associated with the receptor, however previous G protein coupling may be required (69). β-arrestins are also known to activate the mitogen-activated protein kinase pathway, which will influence gene expression (66, 67). The GhrR full agonist ghrelin can activate all these pathways, but it is unknown whether ghrelin activates all pathways to a similar degree in different cell types and tissues (64, 70, 71). Furthermore, the GhrR has a very high and physiologically relevant constitutive activity of approximately 50% of its maximum capacity and it is able to heterodimerize with other GPCRs such as both DA receptor types (70, 72–74). This is particularly interesting as the high constitutive GhrR activity and the heterodimerization potential may be crucial for GhrR signaling in the VTA, as it is still controversial whether peripheral ghrelin can reach the central nervous system (54).

Even though the complexity of GhrR signaling is well established, still very little is known about physiologically relevant actions and pathways of the GhrR in the DAergic system, even though many studies have shown the importance of the GhrR in reward-related behavior [e.g. (75–77)]. Furthermore, only very few studies have attempted to translate the different pathways into physiological functions. This is an obstacle when it comes to designing GhrR targeting drugs as a high pleiotropic signaling could mediate a higher risk of developing adverse effects. Understanding the exact physiological effects of signaling *via* the different GhrR pathways would allow the design of biased agonists or antagonists with fewer adverse effects, e.g. in the treatment of AN. It could further open the possibility to target selectively the primary symptoms of AN, such as increase of motivation to eat and food intake in addition to the secondary symptoms, such as impaired gut motility, anxiety and locomotion (**Figure 2**). Being able to target those secondary symptoms is particularly valuable as clinical trials with AN patients have demonstrated that the patients’ mental state can be negatively affected or sensitive to the increase in appetite and food intake induced by GhrR ligands. Having the increase in food intake accompanied by the attenuation of symptoms that affect the subjective well-being of the patients, such as anxiety and impaired gut motility could lead to a higher compliance to the drug within the AN patient population (34).

### 5.3 Altered Ghrelin System in Anorexia Nervosa Patients

As pointed out before, a stable energy balance is important for every living organism and long-term imbalance will lead to changes and impairments of the organism (15). Prolonged fasting induces alterations in the endocrine system, as the body tries to regulate the extreme situation and an increase in systemic ghrelin levels is one of those (78, 79). A systematic meta-analysis showed that all subtypes of AN lead to an increase in ghrelin levels in the acute/fasting state. After receiving treatment, all AN subtypes had decreased ghrelin levels compared to before the treatment, although still increased compared to healthy controls (80). These findings make ghrelin a possible biomarker for AN. Whether the increase in circulating ghrelin is a cause or result of AN is still not understood.

A recent study showed that in female AN patients the decrease in ghrelin levels 30 min after eating breakfast showed a similar pattern of food-induced decrease to the healthy controls. However, the general ghrelin levels were still higher than in healthy controls. Furthermore, a larger percentage decrease in circulating ghrelin after eating (T30-T0) was associated with a larger decrease in the wanting/motivation to eat food (measured with Visual Analog Scales). This effect was only observed in AN patients and not in the healthy controls. Thus, it seems that AN patients are more sensitive to the decrease in ghrelin levels after eating compared to healthy controls (81). Studies investigating AN commonly use healthy controls as their control group, which hinders examining if alterations are AN-specific or due to acute fasting. A recent study investigated the ghrelin levels in young females including AN patients, healthy controls and non-anorexic individuals with food avoidant behavior. This latter group of individuals had the same body mass index as the AN patients, however without the anxiety of weight gain or preoccupation with body shape AN patients have. AN patients demonstrated increased ghrelin levels compared to both control groups. This hints towards a disorder-specific alteration that may be due to differences in the respective motivational drive (82).

That the effects of GhrR signaling show sex-dependent differences has been observed over the last years (83). However, inconsistencies can be found for the food increasing effect of ghrelin. A study observed that female mice were more sensitive to stress-induced restricted food intake and peripheral ghrelin-administration effects including increased food intake, the latter was reduced when the mice were exposed to stress (84). In contrast, another study observed that with ghrelin administered into the hypothalamus, there was no sex-dependent difference in the effect of ghrelin on food intake (85). It was discussed that the sex-dependent differences of ghrelin-effects may be specific for peripherally administered ghrelin and not relevant for centrally administered ghrelin (83). Examining sex-differences of AN in mouse models has been proven to be difficult. With rodent ABA model studies both males and females have been shown to be more prone to develop the AN-like phenotype and no sex-dependent increased vulnerability to the AN-like phenotype was observed in previous studies (86–88). These inconsistencies increase the difficulty to investigate the pathology of sex-differences in AN, which are however an important part of AN, as females are more prone to develop AN and are generally used more for AN studies (2, 89). An important first step would be to include both/all sexes in any study regarding AN, as more data is needed, particularly in regard to animal models.

Particularly interesting is the current discussion of ghrelin as a survival hormone that is responsible for maintaining body weight (BW) in metabolically and psychologically challenging situations. This is considered in the larger context that includes not only food intake regulation, but also glucose homeostasis or effects of chronic stress (90, 91). Studies have shown that under severe caloric restriction a knockout of the GhrR or GOAT leads to an impaired blood glucose maintenance, a stronger decrease in BW and a reduced survival chance (49, 53, 92, 93). These results indicate the importance of the GhrR in blood glucose and BW control under extreme circumstances. Therefore, it has been suggested that the increase in ghrelin levels in AN patients is a survival response to reduce detrimental effects, e.g. BW loss and anxiety (94). This hypothesis is supported by the fact that functional AgRP neurons, which are activated by ghrelin, are important for energy mobilization and survival during the high activity and low energy states responsible for phenotype in the ABA model (95).

## 6 Biased Ghrelin Signaling in the Context of Anorexia Nervosa

**Figure 3:**
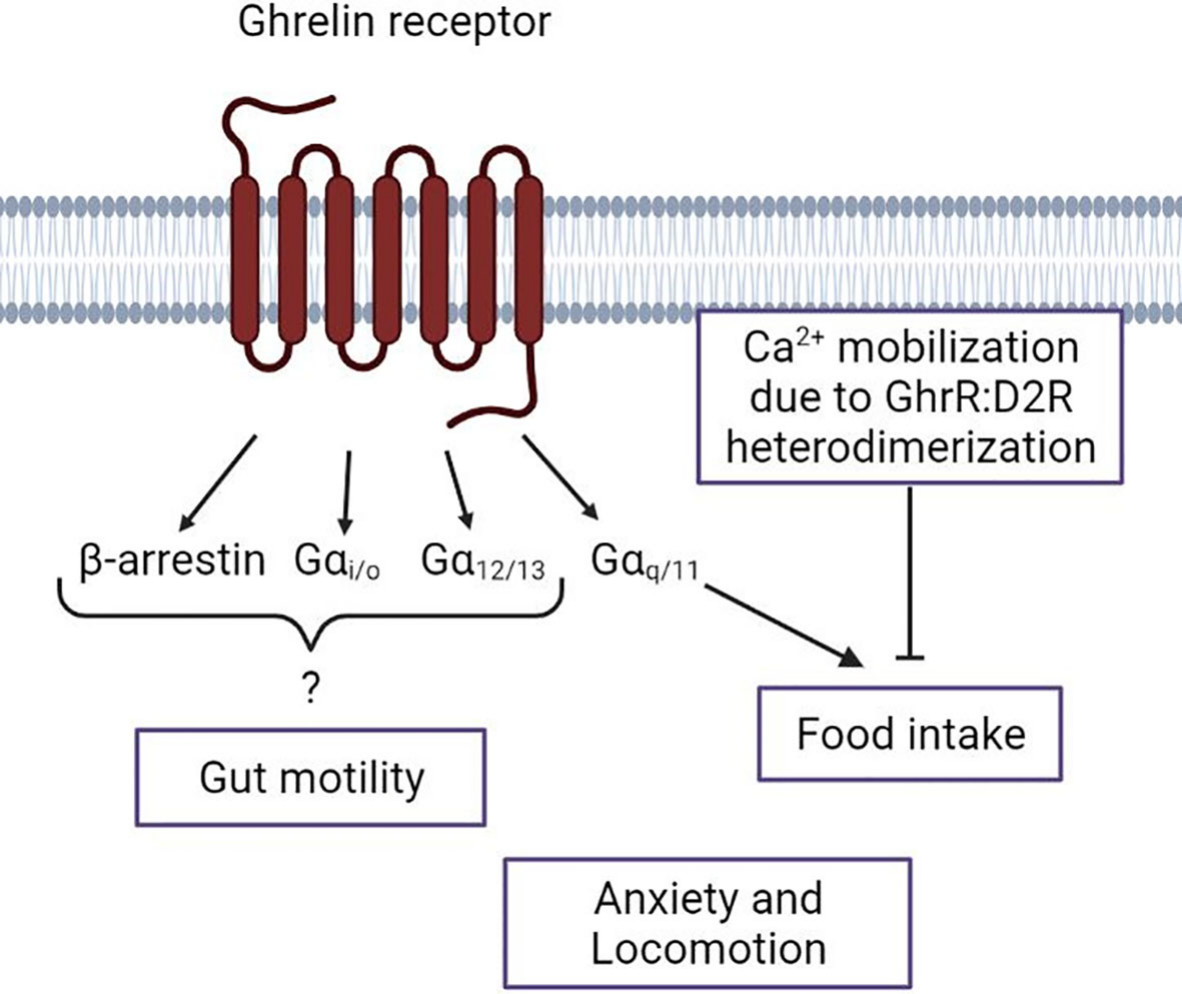
Schematic overview of the ghrelin receptor intracellular signal pathways and their known association with Anorexia Nervosa symptoms. Depiction of the current knowledge of the respective Gα-protein and β-arrestin actions regarding the Anorexia Nervosa-associated ghrelin receptor functions. The lack of a connection of a specific intracellular pathway to anxiety and locomotion. Figure was created with BioRender.com.

A closer understanding of the physiological functions mediated by each of the four GhrR pathways is crucial for a suitable GhrR drug-targeting. A study observed that biased signaling potentially cannot only be found for GhrR agonists or antagonists, but also for inverse agonists, which opens a lot of possibilities due to the high and physiologically relevant constitutive activity of the GhrR (96). Furthermore, many studies in the recent years have provided better insight into the key residues of the GhrR that are important to initiate conformational change due to biased agonist binding (70). This comprehensive knowledge could allow for the design of biased agonists with minor modulations leading to very specific activation pattern of the physiological functions, which could perfectly fit the AN symptom profile and improve drug compliance. In the following section we briefly summarize the current knowledge on the intracellular GhrR signaling pathways in regard to food intake, locomotion, anxiety and gut motility (**Figure 3**).

### 6.1 Food Intake

Ghrelin is the only known peptide hormone from the gut that has an orexigenic effect (54). The most extensively studied orexigenic ghrelin activity is *via* the homeostatic system. Ghrelin signaling can reach the hypothalamus both *via* vagal innervation and *via* the circulation. It is well established that endocrine ghrelin binds to GhrRs on NPY/AgRP neurons in the ARC, activates them and induces their orexigenic effects, which can lead to increased food intake and adiposity (97–99).

Regarding transgenic ghrelin- or GhrR-deficiency models, no big effect on food intake or BW can usually be observed under basal conditions. However, when the model is metabolically challenged, the importance of the ghrelin pathway can often be observed. For instance, it has been shown that transgenic mice with knockout of the GhrR have a decreased calorie consumption when on a HFD, compared to wild type mice (100). As most studies were performed with male mice, sex-dependent differences of food intake in GhrR knockout mice are only recently explored. It was demonstrated that in males the reduction of mean meal number due to a GhrR knockout was significant while in females no significant mean meal number reduction was observed. However, other food-related parameters showed no genotype x sex interaction effect (101). In one study, a mouse model with a selective knockout of the Gαq/11 subunits in AgRP neurons was generated to gain an insight on the relevance of this specific ghrelin-activated pathway in the context of appetite regulation. The study showed that under basal conditions no difference in food intake could be seen in the knockout mice compared to wild type mice. However, with intracerebroventricular (ICV) administration of ghrelin both knockout and wild type mice showed a significant increase in food intake 60min after administration. Nevertheless, ghrelin-induced food intake of the knockout mice was significantly reduced compared to the food intake in the wild type mice. This indicates the importance of the Gαq/11 pathway in ARC AgRP neurons in ghrelin-induced food intake (**Figure 3**). The importance of the Gαq/11 pathway was further supported with the biased GhrR agonists Abb13d and YIL781. The inverse agonist for Gαq/11 signaling, Abb13d led to a decrease in food intake during dark phase. The partial agonist YIL781 led to no difference during dark phase, but to an increase in food intake during light phase. This demonstrates that even a partial Gαq/11 activation can induce an increase in food intake, at least during light phase when endogenous ghrelin is low, whereas an inhibition of the Gαq/11 pathway in AgRP neurons will result in reduced food intake (62).

### 6.2 Locomotion

Excessive locomotion in the form of overexercising is a pivotal part of AN, particularly in the restricted type (35). Ghrelin has been shown to be important in the anticipatory increase in locomotion for scheduled meals in rodents. The anticipatory locomotion is positively correlated with ghrelin plasma levels in ABA rats. The same study looked at the differences in anticipatory behavior of ABA rats after GhrR antagonist treatments or in GhrR knockouts. Both the antagonists and knockout of the GhrR led to a decrease in anticipatory activity (102). Contradictory, ghrelin knockout mice fed with a high-fat diet (HFD) had an increase in energy expenditure and locomotor activity compared to wild type mice, which indicates a different effect of ghrelin and GhrR signaling depending on the metabolic state (103). Investigating how GhrR signaling is involved in increased locomotion, particularly in the VTA, may contribute to understanding the etiology of AN. Furthermore, when designing a potential AN drug targeting the GhrR a further increase of locomotion and therefore energy expenditure in the patients could counteract the therapy.

### 6.3 Anxiety

That ghrelin plays a role in anxiety regulation has been shown in several studies, however with contradictory results; both anxiolytic and anxiogenic properties have been attributed to GhrR signaling (104–106). All these studies have been performed under slightly different conditions, which could imply that differently modulated GhrR pathways were initiated. Results from a study provided insight on the importance of GhrR expression sites in anxiety modulation. The study demonstrated a general anxiolytic effect of high peripheral ghrelin doses. Additionally, the authors found that an overexpression of the GhrR led to a weak or strong anxiolytic effect, depending on the expression level in the lateral nuclei of the amygdala, which could indicate that GhrR effects on anxiety depend on the nuclei in which it is expressed (58). However, no study has connected the anxiety-modulating effects to a specific GhrR pathway. Examining which of the GhrR signaling pathways are involved in the anxiety-modulating effects may help to understand the contradictory results so far. The possibility of translating a GhrR pathway to the potential anxiolytic effects of ghrelin may allow a reduction of anxiety in AN patients with a GhrR targeting drug. Furthermore, it could help to investigate if GhrR:DA receptor heterodimerization occurs in the amygdala and which DA receptor types and pathways are involved.

### 6.4 Gut Motility

AN patients suffer from an impairment in gut motility, which is reported as one of the main factors reducing the quality of life (107, 108). GhrR signaling in the gastrointestinal tract leads to increased gastric emptying/gut motility (109). In a GhrR biased signaling translation study, two GhrR agonists were used with different properties; Abb13d is an inverse agonist for Gαq/11 and YIL781 is a partial agonist for the same pathway. Both agonists do not or barely activate other GhrR pathways, including β-arrestin recruitment. Both biased agonists led to a decrease in gastric emptying compared to vehicle administration, which indicates that the Gαq/11 pathway is not responsible for inducing gastric emptying/gut motility *via* the GhrR (62) (**Figure 3**). It was observed that administration of YIL781 also interferes with defecation induction of intrathecal administered DA. This study further demonstrated a GhrR and D2R co-expression in autonomic neurons. As it is generally assumed that ghrelin is not reaching the central nervous system, these results suggest that a GhrR and D2R signaling interaction, potentially heterodimerization, is necessary for DA-induced defecation (110).

## 7 Ghrelin Receptor Signaling in the Dopaminergic System

When intending to target the GhrR pathways in the context of AN, it is important to take the DAergic system into account. The importance of ghrelin in reward-related behavior, which is modulated by the DAergic system, has been extensively reviewed [e.g. (7, 55, 111)]. The DA-meditated motivation to obtain food is crucial for food intake, as it has been highlighted in section 2.1.2. However, D1R- and D2R-like pathways have opposing effects in in their role of food intake. Peripheral administered D1R antagonists have been shown to decrease food-seeking behavior, while D2R antagonist or knockouts enhance food-seeking behavior (112). A functional GhrR signaling is pivotal for the food-seeking regulation of the DAergic system. It was observed that a selective D2R agonist that induces anorexia in wild type mice was incapable of inducing anorexia in GhrR^-/-^ mice. In contrast when only the ligand was missing in ghrelin^-/-^ mice, anorexia was induced by the D2R agonist. With further examination it was demonstrated that interfering with GhrR:D2R heterodimerization, which was found most abundantly in the hypothalamus, led to an inhibition of the anorexia-inducing effect of the D2R agonist. Further, it was observed that GhrR:D2R heterodimerization led to the activation of the Gβγ-coupled induction of Ca^2+^ mobilization, instead of the standard Gαi/o-coupled pathway of D2R (72) (**Figure 3**). When reintroducing the GhrR only into the VTA in a GhrR^‐/-^ model, no difference in food intake was seen under basal conditions. However, when exposed to novel environment stress the mice with the VTA-only GhrR expression showed an increase in food intake compared to GhrR^-/-^ mice. Furthermore, the VTA-only GhrR mice were more responsive to cocaine-induced effects compared to the GhrR^-/-^ mice. These results indicate the importance of the GhrR with regards to reward-related and stress response coping behavior. The study further demonstrated the importance of the GhrR in the VTA for DA-induced locomotion. GhrR expression in the VTA was sufficient to increase cocaine-induced hyperlocomotion compared to the global GhrR knockout mice (113). A recent study has demonstrated with a GhrR knockout and GhrR antagonist, both specifically impairing GhrR signaling in the VTA of male mice, a reduced social motivation (114). These findings are particularly interesting as social anxiety is often associated with eating disorders (115). This makes GhrR signaling in the VTA a promising target for understanding the complex role of ghrelin and the DAergic mesolimbic system in AN.

We highlighted the interconnective potential of GhrR signaling and the DAergic system. However, very little is understood on how the different pathways of the GhrR might induce different signaling patterns in the DAergic system. Various studies have demonstrated that ghrelin- or GhrR-induced effects vary depending on environmental, psychological, or metabolic state (7, 28, 84, 113). This combined with the hypothesis of ghrelin as a survival hormone suggests, that the modulatory effects of ghrelin and GhrR signaling are capable to adapt to different situations. As the metabolic state changes quite drastically throughout the course of AN it is possible that an altered activation of the GhrR affects the signaling in the DAergic system and therefore the motivational drive of the patients. That microenvironmental changes can affect GhrR signaling patterns, is supported by a recent study observing that lipid membrane composition and thickness have the potential to alter the GhrR activation and conformational state (116). Altered lipid composition and integrity of cell membranes have been observed in AN patients (117, 118). This could be a potential way how alterations of the intracellular GhrR signaling pathways in the DAergic system could be part of or accelerate the AN development. However, this is speculative and extensive research is needed to understand the underlying mechanisms of AN. Future studies should aim at investigating the effects of peripherally administered biased GhrR ligands, to explore the clinical potential of biased GhrR ligands as AN treatment. Furthermore, the modulatory effect of GhrR signaling in the DAergic system as part of the AN etiology has to be explored more extensively, by examining intracellular and cellular activation patterns altered in different e.g. food intake and reward-related situations with subjects in different environmental, psychological and metabolic states. This implies not only the different signal pathways, but also the heterodimerization potential of the receptors.

## 8 Conclusion

This review aims to highlight two main conclusions. First, a biased agonist of the GhrR may constitute a promising target for AN treatment, however extensive and systematic translational studies are needed. Second, targeting the GhrR must be performed with consideration of the DAergic system as GhrR signaling and DA signaling are closely associated in the context of AN. In conclusion, regardless of the limitations and complexity in this research area, the close functional and anatomical connection of GhrR and DA signaling is a promising field to explore in the context of AN. In particular, increased understanding of the close integration and the signaling properties of the ghrelin and the dopaminergic pathways may mature this “receptor-couple” into a fruitful target in the treatment of AN.

## Author Contributions

MSK wrote the review with input from all authors. All authors contributed to the article and approved the submitted version.

## Funding

The Lundbeck Foundation Ascending Investigators grant (R344-2020-810)– To study “Biased ligands for the ghrelin receptor as treatment for Anorexia Nervosa” and The Novo Nordisk Foundation - Project grants in Endocrinology and Metabolism – Nordic Region (0057417).

## Conflict of Interest

The authors declare that the research was conducted in the absence of any commercial or financial relationships that could be construed as a potential conflict of interest.

## Publisher’s Note

All claims expressed in this article are solely those of the authors and do not necessarily represent those of their affiliated organizations, or those of the publisher, the editors and the reviewers. Any product that may be evaluated in this article, or claim that may be made by its manufacturer, is not guaranteed or endorsed by the publisher.

## References

[1] Chidiac CW. An Update on the Medical Consequences of Anorexia Nervosa. Curr Opin Pediatr (2019) 31:448–53. doi: 10.1097/MOP.0000000000000755 30883398

[2] Chaer R Nakouzi N Itani L Tannir H Kreidieh D el Masri D. Fertility and Reproduction After Recovery From Anorexia Nervosa: A Systematic Review and Meta-Analysis of Long-Term Follow-Up Studies. Dis (Basel Switzerland) (2020) 8:46. doi: 10.3390/diseases8040046 PMC7768504 33339411

[3] Blanchet C Guillaume S Bat-Pitault F Carles M-E Clarke J Dodin V. Medication in AN: A Multidisciplinary Overview of Meta-Analyses and Systematic Reviews. J Clin Med (2019) 8:278. doi: 10.3390/jcm8020278 PMC6406645 30823566

[4] Méquinion M Foldi CJ Andrews ZB. The Ghrelin-AgRP Neuron Nexus in Anorexia Nervosa: Implications for Metabolic and Behavioral Adaptations. Front Nutr (2019) 6:190. doi: 10.3389/fnut.2019.00190 31998738 PMC6962137

[5] Gorwood P Blanchet-Collet C Chartrel N Duclos J Dechelotte P Hanachi M. New Insights in Anorexia Nervosa. Front Neurosci (2016) 10:256. doi: 10.3389/fnins.2016.00256 27445651 PMC4925664

[6] Forrest LN Jones PJ Ortiz SN Smith AR. Core Psychopathology in Anorexia Nervosa and Bulimia Nervosa: A Network Analysis. Int J Eat Disord (2018) 51:668–79. doi: 10.1002/eat.22871 29693747

[7] Al Massadi O Nogueiras R Dieguez C Girault J-A. Ghrelin and Food Reward. Neuropharmacology (2019) 148:131–8. doi: 10.1016/j.neuropharm.2019.01.001 30615902

[8] Stievenard A Méquinion M Andrews ZB Destée A Chartier-Harlin M-C Viltart O. Is There a Role for Ghrelin in Central Dopaminergic Systems? Focus on Nigrostriatal and Mesocorticolimbic Pathways. Neurosci Biobehav Rev (2017) 73:255–75. doi: 10.1016/j.neubiorev.2016.11.021 27914942

[9] Jiang T Soussignan R Carrier E Royet J-P. Dysfunction of the Mesolimbic Circuit to Food Odors in Women With Anorexia and Bulimia Nervosa: A fMRI Study. Front Hum Neurosci (2019) 13:117. doi: 10.3389/fnhum.2019.00117 31019456 PMC6458263

[10] Berner LA Brown TA Lavender JM Lopez E Wierenga CE Kaye WH. Neuroendocrinology of Reward in Anorexia Nervosa and Bulimia Nervosa: Beyond Leptin and Ghrelin. Mol Cell Endocrinol (2019) 497:110320. doi: 10.1016/j.mce.2018.10.018 30395874 PMC6497565

[11] Foldi CJ Milton LK Oldfield BJ. The Role of Mesolimbic Reward Neurocircuitry in Prevention and Rescue of the Activity-Based Anorexia (ABA) Phenotype in Rats. Neuropsychopharmacol: Off Publ Am Coll Neuropsychopharmacol (2017) 42:2292–300. doi: 10.1038/npp.2017.63 PMC5645746 28322236

[12] Gurevich VV Gurevich EV. Biased GPCR Signaling: Possible Mechanisms and Inherent Limitations. Pharmacol Ther (2020) 211:107540. doi: 10.1016/j.pharmthera.2020.107540 32201315 PMC7275904

[13] World Health Organization. Obesity and Overweight. World Health Organization (2020). Available at: https://www.who.int/news-room/fact-sheets/detail/obesity-and-overweight

[14] Baracos VE Martin L Korc M Guttridge DC Fearon KCH. Cancer-Associated Cachexia. Nat Rev Dis Primers (2018) 4:17105. doi: 10.1038/nrdp.2017.105 29345251

[15] Faulconbridge LF Hayes MR. Regulation of Energy Balance and Body Weight by the Brain: A Distributed System Prone to Disruption. Psychiatr Clin North Am (2011) 34:733–45. doi: 10.1016/j.psc.2011.08.008 PMC3222868 22098800

[16] Liu CM Kanoski SE. Homeostatic and non-Homeostatic Controls of Feeding Behavior: Distinct vs. Common Neural Systems. Physiol Behav (2018) 193:223–31. doi: 10.1016/j.physbeh.2018.02.011 PMC6077115 29421588

[17] Schwartz MW Woods SC Seeley RJ Barsh GS Baskin DG Leibel RL. Is the Energy Homeostasis System Inherently Biased Toward Weight Gain? Diabetes (2003) 52:232–8. doi: 10.2337/diabetes.52.2.232 12540591

[18] Szczypka MS Rainey MA Kim DS Alaynick WA Marck BT Matsumoto AM. Feeding Behavior in Dopamine-Deficient Mice. Proc Natl Acad Sci USA (1999) 96:12138–43. doi: 10.1073/pnas.96.21.12138 PMC18425 10518589

[19] Palmiter RD. Is Dopamine a Physiologically Relevant Mediator of Feeding Behavior? Trends Neurosci (2007) 30:375–81. doi: 10.1016/j.tins.2007.06.004 17604133

[20] Abizaid A Liu Z-W Andrews ZB Shanabrough M Borok E Elsworth JD. Ghrelin Modulates the Activity and Synaptic Input Organization of Midbrain Dopamine Neurons While Promoting Appetite. J Clin Invest (2006) 116:3229–39. doi: 10.1172/JCI29867 PMC1618869 17060947

[21] Merchenthaler I Lane M Shughrue P. Distribution of Pre-Pro-Glucagon and Glucagon-Like Peptide-1 Receptor Messenger RNAs in the Rat Central Nervous System. J Comp Neurol (1999) 403:261–80. doi: 10.1002/(SICI)1096-9861(19990111)403:2<261::AID-CNE8>3.0.CO;2-5 9886047

[22] Figlewicz DP Evans SB Murphy J Hoen M Baskin DG. Expression of Receptors for Insulin and Leptin in the Ventral Tegmental Area/Substantia Nigra (VTA/SN) of the Rat. Brain Res (2003) 964:107–15. doi: 10.1016/S0006-8993(02)04087-8 12573518

[23] Leinninger GM Opland DM Jo Y-H Faouzi M Christensen L Cappellucci LA. Leptin Action *via* Neurotensin Neurons Controls Orexin, the Mesolimbic Dopamine System and Energy Balance. Cell Metab (2011) 14:313–23. doi: 10.1016/j.cmet.2011.06.016 PMC3183584 21907138

[24] Larsen PJ Tang-Christensen M Holst JJ Ørskov C. Distribution of Glucagon-Like Peptide-1 and Other Preproglucagon-Derived Peptides in the Rat Hypothalamus and Brainstem. Neuroscience (1997) 77:257–70. doi: 10.1016/S0306-4522(96)00434-4 9044391

[25] Cornejo MP Barrile F Francesco PN Portiansky EL Reynaldo M Perello M. Ghrelin Recruits Specific Subsets of Dopamine and GABA Neurons of Different Ventral Tegmental Area Sub-Nuclei. Neuroscience (2018) 392:107–20. doi: 10.1016/j.neuroscience.2018.09.027 30268780

[26] Asakawa A Inui A Kaga T Yuzuriha H Nagata T Fujimiya M. A Role of Ghrelin in Neuroendocrine and Behavioral Responses to Stress in Mice. Neuroendocrinology (2001) 74:143–7. doi: 10.1159/000054680 11528215

[27] Mondal MS Date Y Yamaguchi H Toshinai K Tsuruta T Kangawa K. Identification of Ghrelin and its Receptor in Neurons of the Rat Arcuate Nucleus. Regul Pept (2005) 126:55–9. doi: 10.1016/j.regpep.2004.08.038 15620414

[28] Kawahara Y Kawahara H Kaneko F Yamada M Nishi Y Tanaka E. Peripherally Administered Ghrelin Induces Bimodal Effects on the Mesolimbic Dopamine System Depending on Food-Consumptive States. Neuroscience (2009) 161:855–64. doi: 10.1016/j.neuroscience.2009.03.086 19362120

[29] Edvardsson CE Vestlund J Jerlhag E. A Ghrelin Receptor Antagonist Reduces the Ability of Ghrelin, Alcohol or Amphetamine to Induce a Dopamine Release in the Ventral Tegmental Area and in Nucleus Accumbens Shell in Rats. Eur J Pharmacol (2021) 899:174039. doi: 10.1016/j.ejphar.2021.174039 33737011

[30] Castro DC Cole SL Berridge KC. Lateral Hypothalamus, Nucleus Accumbens, and Ventral Pallidum Roles in Eating and Hunger: Interactions Between Homeostatic and Reward Circuitry. Front Syst Neurosci (2015) 9:90. doi: 10.3389/fnsys.2015.00090 26124708 PMC4466441

[31] Dietrich MO Bober J Ferreira JG Tellez LA Mineur YS Souza DO. AgRP Neurons Regulate Development of Dopamine Neuronal Plasticity and Nonfood-Associated Behaviors. Nat Neurosci (2012) 15:1108–10. doi: 10.1038/nn.3147 PMC3411867 22729177

[32] Rikani AA Choudhry Z Maqsood Choudhry A Ikram H Waheed Asghar M Kajal D. A Critique of the Literature on Etiology of Eating Disorders. Ann Neurosci (2013) 20:157–61. doi: 10.5214/ans.0972.7531.200409 PMC4117136 25206042

[33] Watson HJ Yilmaz Z Thornton LM Hübel C Coleman JRI Gaspar HA. Genome-Wide Association Study Identifies Eight Risk Loci and Implicates Metabo-Psychiatric Origins for Anorexia Nervosa. Nat Genet (2019) 51:1207–14. doi: 10.1038/s41588-019-0439-2 PMC6779477 31308545

[34] Halmi KA. The Perplexities of Conducting Randomized, Double-Blind, Placebo-Controlled Treatment Trials in Anorexia Nervosa Patients. Am J Psychiatry (2008) 165:1227–8. doi: 10.1176/appi.ajp.2008.08060957 18829874

[35] Dalle Grave R Calugi S Marchesini G. Compulsive Exercise to Control Shape or Weight in Eating Disorders: Prevalence, Associated Features, and Treatment Outcome. Compr Psychiatry (2008) 49:346–52. doi: 10.1016/j.comppsych.2007.12.007 18555054

[36] Kim SF. Animal Models of Eating Disorders. Neuroscience (2012) 211:2–12. doi: 10.1016/j.neuroscience.2012.03.024 22465439 PMC3351502

[37] Wise RA. Dopamine, Learning and Motivation. Nat Rev Neurosci (2004) 5:483–94. doi: 10.1038/nrn1406 15152198

[38] Miller JD Sanghera MK German DC. Mesencephalic Dopaminergic Unit Activity in the Behaviorally Conditioned Rat. Life Sci (1981) 29:1255–63. doi: 10.1016/0024-3205(81)90231-9 7300554

[39] Glavin GB Szabo S. Dopamine in Gastrointestinal Disease. Dig Dis Sci (1990) 35:1153–61. doi: 10.1007/BF01537589 2202571

[40] Greba Q Gifkins A Kokkinidis L. Inhibition of Amygdaloid Dopamine D2 Receptors Impairs Emotional Learning Measured With Fear-Potentiated Startle. Brain Res (2001) 899:218–26. doi: 10.1016/S0006-8993(01)02243-0 11311883

[41] Deutch AY Tam S-Y Roth RH. Footshock and Conditioned Stress Increase 3, 4-Dihydroxyphenylacetic Acid (DOPAC) in the Ventral Tegmental Area But Not Substantia Nigra. Brain Res (1985) 333:143–6. doi: 10.1016/0006-8993(85)90134-9 3995282

[42] Kravitz AV Freeze BS Parker PRL Kay K Thwin MT Deisseroth K. Regulation of Parkinsonian Motor Behaviours by Optogenetic Control of Basal Ganglia Circuitry. Nature (2010) 466:622–6. doi: 10.1038/nature09159 PMC3552484 20613723

[43] Free RB Chun LS Moritz AE Miller BN Doyle TB Conroy JL. Discovery and Characterization of a G Protein–Biased Agonist That Inhibits *β* -Arrestin Recruitment to the D2 Dopamine Receptor. Mol Pharmacol (2014) 86:95–105. doi: 10.1124/mol.113.090563 PMC4054005 24755247

[44] Mei C Ramos M Iitaka C Borrelli E. Getting Specialized: Presynaptic and Postsynaptic Dopamine D2 Receptors. Curr Opin Pharmacol (2009) 9:53–8. doi: 10.1016/j.coph.2008.12.002 PMC2710814 19138563

[45] Harrison A O’Brien N Lopez C Treasure J. Sensitivity to Reward and Punishment in Eating Disorders. Psychiatry Res (2010) 177:1–11. doi: 10.1016/j.psychres.2009.06.010 20381877

[46] DeGuzman M Shott ME Yang TT Riederer J Frank GKW. Association of Elevated Reward Prediction Error Response With Weight Gain in Adolescent Anorexia Nervosa. Am J Psychiatry (2017) 174:557–65. doi: 10.1176/appi.ajp.2016.16060671 PMC5607032 28231717

[47] Frank GKW Shott ME Stoddard J Swindle S Pryor TL. Association of Brain Reward Response With Body Mass Index and Ventral Striatal-Hypothalamic Circuitry Among Young Women With Eating Disorders. JAMA Psychiatry (2021). doi: 10.1001/jamapsychiatry.2021.1580 PMC8246338 34190963

[48] Beeler JA Mourra D Zanca RM Kalmbach A Gellman C Klein BY. Vulnerable and Resilient Phenotypes in a Mouse Model of Anorexia Nervosa. Biol Psychiatry (2020). doi: 10.1016/j.biopsych.2020.06.030 PMC7855473 32950210

[49] Kojima M Hosoda H Date Y Nakazato M Matsuo H Kangawa K. Ghrelin is a Growth-Hormone-Releasing Acylated Peptide From Stomach. Nature (1999) 402:656–60. doi: 10.1038/45230 10604470

[50] Gnanapavan S Kola B Bustin SA Morris DG McGee P Fairclough P. The Tissue Distribution of the mRNA of Ghrelin and Subtypes of its Receptor, GHS-R, in Humans. J Clin Endocrinol Metab (2002) 87:2988. doi: 10.1210/jcem.87.6.8739 12050285

[51] Date Y Kojima M Hosoda H Sawaguchi A Mondal MS Suganuma T. Ghrelin, a Novel Growth Hormone-Releasing Acylated Peptide, is Synthesized in a Distinct Endocrine Cell Type in the Gastrointestinal Tracts of Rats and Humans. Endocrinology (2000) 141:4255–61. doi: 10.1210/endo.141.11.7757 11089560

[52] Gutierrez JA Solenberg PJ Perkins DR Willency JA Knierman MD Jin Z. Ghrelin Octanoylation Mediated by an Orphan Lipid Transferase. Proc Natl Acad Sci (2008) 105:632–5. doi: 10.1073/pnas.0800708105 PMC2359796 18443287

[53] Yang J Brown MS Liang G Grishin NV Goldstein JL. Identification of the Acyltransferase That Octanoylates Ghrelin, an Appetite-Stimulating Peptide Hormone. Cell (2008) 132:387–96. doi: 10.1016/j.cell.2008.01.017 18267071

[54] Müller TD Nogueiras R Andermann ML Andrews ZB Anker SD Argente J. Ghrelin. Mol Metab (2015) 4:437–60. doi: 10.1016/j.molmet.2015.03.005 PMC4443295 26042199

[55] Perello M Dickson SL. Ghrelin Signalling on Food Reward: A Salient Link Between the Gut and the Mesolimbic System. J Neuroendocrinol (2015) 27:424–34. doi: 10.1111/jne.12236 PMC5033008 25377898

[56] da Fonseca GWP von Haehling S. An Overview of Anamorelin as a Treatment Option for Cancer-Associated Anorexia and Cachexia. Expert Opin Pharmacother (2021) 22:889–95. doi: 10.1080/14656566.2021.1873954 33491505

[57] Liu H Zang P Lee II-g Anderson B Christiani A Strait-Bodey L. Growth Hormone Secretagogue Receptor-1a Mediates Ghrelin’s Effects on Attenuating Tumour-Induced Loss of Muscle Strength But Not Muscle Mass. J Cachexia Sarcopenia Muscle (2021). doi: 10.1002/jcsm.12743 PMC8517358 34264027

[58] Jensen M Ratner C Rudenko O Christiansen SH Skov LJ Hundahl C. Anxiolytic-Like Effects of Increased Ghrelin Receptor Signaling in the Amygdala. Int J Neuropsychopharmacol (2016) 19:pyv123. doi: 10.1093/ijnp/pyv123 26578081 PMC4886665

[59] Zigman JM Jones JE Lee CE Saper CB Elmquist JK. Expression of Ghrelin Receptor mRNA in the Rat and the Mouse Brain. J Comp Neurol (2006) 494:528–48. doi: 10.1002/cne.20823 PMC4524499 16320257

[60] Guan X-M Yu H Palyha OC McKee KK Feighner SD Sirinathsinghji DJS. Distribution of mRNA Encoding the Growth Hormone Secretagogue Receptor in Brain and Peripheral Tissues. Mol Brain Res (1997) 48:23–9. doi: 10.1016/S0169-328X(97)00071-5 9379845

[61] Davies MN Secker A Freitas AA Mendao M Timmis J Flower DR. On the Hierarchical Classification of G Protein-Coupled Receptors. Bioinformatics (2007) 23:3113–8. doi: 10.1093/bioinformatics/btm506 17956878

[62] Mende F Hundahl C Plouffe B Skov LJ Sivertsen B Madsen AN. Translating Biased Signaling in the Ghrelin Receptor System Into Differential *In Vivo* Functions. Proc Natl Acad Sci USA (2018) 115:E10255–64. doi: 10.1073/pnas.1804003115 PMC6205473 30301804

[63] Howard AD Feighner SD Cully DF Arena JP Liberator PA Rosenblum CI. A Receptor in Pituitary and Hypothalamus That Functions in Growth Hormone Release. Sci (New York NY) (1996) 273:974–7. doi: 10.1126/science.273.5277.974 8688086

[64] Holst B Cygankiewicz A Jensen TH Ankersen M Schwartz TW. High Constitutive Signaling of the Ghrelin Receptor–Identification of a Potent Inverse Agonist. Mol Endocrinol (Baltimore Md) (2003) 17:2201–10. doi: 10.1210/me.2003-0069 12907757

[65] Holst B Lang M Brandt E Bach A Howard A Frimurer TM. Ghrelin Receptor Inverse Agonists: Identification of an Active Peptide Core and its Interaction Epitopes on the Receptor. Mol Pharmacol (2006) 70:936–46. doi: 10.1124/mol.106.024422 16798937

[66] Neves SR Ram PT Iyengar R. G Protein Pathways. Science (2002) 296:1636–9. doi: 10.1126/science.1071550 12040175

[67] Hilger D Masureel M Kobilka BK. Structure and Dynamics of GPCR Signaling Complexes. Nat Struct Mol Biol (2018) 25:4–12. doi: 10.1038/s41594-017-0011-7 29323277 PMC6535338

[68] Riobo NA Manning DR. Receptors Coupled to Heterotrimeric G Proteins of the G12 Family. Trends Pharmacol Sci (2005) 26:146–54. doi: 10.1016/j.tips.2005.01.007 15749160

[69] Grundmann M Merten N Malfacini D Inoue A Preis P Simon K. Lack of Beta-Arrestin Signaling in the Absence of Active G Proteins. Nat Commun (2018) 9:341. doi: 10.1038/s41467-017-02661-3 29362459 PMC5780443

[70] Hedegaard MA Holst B. The Complex Signaling Pathways of the Ghrelin Receptor. Endocrinology (2020) 161:bqaa020. doi: 10.1210/endocr/bqaa020 32049280

[71] Damian M Marie J Leyris J-P Fehrentz J-A Verdié P Martinez J. High Constitutive Activity is an Intrinsic Feature of Ghrelin Receptor Protein: A Study With a Functional Monomeric GHS-R1a Receptor Reconstituted in Lipid Discs. J Biol Chem (2012) 287:3630–41. doi: 10.1074/jbc.M111.288324 PMC3281683 22117076

[72] Kern A Albarran-Zeckler R Walsh HE Smith RG. Apo-Ghrelin Receptor Forms Heteromers With DRD2 in Hypothalamic Neurons and Is Essential for Anorexigenic Effects of DRD2 Agonism. Neuron (2012) 73:317–32. doi: 10.1016/j.neuron.2011.10.038 PMC3269786 22284186

[73] Jiang H Betancourt L Smith RG. Ghrelin Amplifies Dopamine Signaling by Cross Talk Involving Formation of Growth Hormone Secretagogue Receptor/Dopamine Receptor Subtype 1 Heterodimers. Mol Endocrinol (Baltimore Md) (2006) 20:1772–85. doi: 10.1210/me.2005-0084 16601073

[74] Holliday ND Holst B Rodionova EA Schwartz TW Cox HM. Importance of Constitutive Activity and Arrestin-Independent Mechanisms for Intracellular Trafficking of the Ghrelin Receptor. Mol Endocrinol (Baltimore Md) (2007) 21:3100–12. doi: 10.1210/me.2007-0254 17717076

[75] Perello M Sakata I Birnbaum S Chuang J-C Osborne-Lawrence S Rovinsky SA. Ghrelin Increases the Rewarding Value of High-Fat Diet in an Orexin-Dependent Manner. Biol Psychiatry (2010) 67:880–6. doi: 10.1016/j.biopsych.2009.10.030 PMC2854245 20034618

[76] Egecioglu E Jerlhag E Salomé N Skibicka KP Haage D Bohlooly--M. Ghrelin Increases Intake of Rewarding Food in Rodents. Addict Biol (2010) 15:304–11. doi: 10.1111/j.1369-1600.2010.00216.x PMC2901520 20477752

[77] Malik S McGlone F Bedrossian D Dagher A. Ghrelin Modulates Brain Activity in Areas That Control Appetitive Behavior. Cell Metab (2008) 7:400–9. doi: 10.1016/j.cmet.2008.03.007 18460331

[78] Kroemer NB Krebs L Kobiella A Grimm O Pilhatsch M Bidlingmaier M. Fasting Levels of Ghrelin Covary With the Brain Response to Food Pictures. Addict Biol (2013) 18:855–62. doi: 10.1111/j.1369-1600.2012.00489.x 22974271

[79] Shiiya T Nakazato M Mizuta M Date Y Mondal MS Tanaka M. Plasma Ghrelin Levels in Lean and Obese Humans and the Effect of Glucose on Ghrelin Secretion. J Clin Endocrinol Metab (2002) 87:240–4. doi: 10.1210/jcem.87.1.8129 11788653

[80] Seidel M Markmann Jensen S Healy D Dureja A Watson HJ Holst B. A Systematic Review and Meta-Analysis Finds Increased Blood Levels of All Forms of Ghrelin in Both Restricting and Binge-Eating/Purging Subtypes of Anorexia Nervosa. Nutrients (2021) 13:709. doi: 10.3390/nu13020709 33672297 PMC7926807

[81] Mancuso C Izquierdo A Slattery M Becker KR Plessow F Thomas JJ. Changes in Appetite-Regulating Hormones Following Food Intake Are Associated With Changes in Reported Appetite and a Measure of Hedonic Eating in Girls and Young Women With Anorexia Nervosa. Psychoneuroendocrinology (2020) 113:104556. doi: 10.1016/j.psyneuen.2019.104556 31918391 PMC7080573

[82] Becker KR Mancuso C Dreier MJ Asanza E Breithaupt L Slattery M. Ghrelin and PYY in Low-Weight Females With Avoidant/Restrictive Food Intake Disorder Compared to Anorexia Nervosa and Healthy Controls. Psychoneuroendocrinology (2021) 129:105243. doi: 10.1016/j.psyneuen.2021.105243 34049199 PMC8363304

[83] Yamada C. Relationship Between Orexigenic Peptide Ghrelin Signal, Gender Difference and Disease. Int J Mol Sci (2021) 22:3763. doi: 10.3390/ijms22073763 33916403 PMC8038632

[84] Yamada C Iizuka S Nahata M Hattori T Takeda H. Vulnerability to Psychological Stress-Induced Anorexia in Female Mice Depends on Blockade of Ghrelin Signal in Nucleus Tractus Solitarius. Br J Pharmacol (2020) 177:4666–82. doi: 10.1111/bph.15219 PMC7520439 32754963

[85] Currie PJ Mirza A Fuld R Park D Vasselli JR. Ghrelin is an Orexigenic and Metabolic Signaling Peptide in the Arcuate and Paraventricular Nuclei. Am J Physiol Regul Integr Comp Physiol (2005) 289:R353–8. doi: 10.1152/ajpregu.00756.2004 15817841

[86] Boakes RA Mills KJ Single JP. Sex Differences in the Relationship Between Activity and Weight Loss in the Rat. Behav Neurosci (1999) 113:1080–9. doi: 10.1037/0735-7044.113.5.1080 10571490

[87] Paré WP Vincent GP Isom KE Reeves JM. Sex Differences and Incidence of Activity-Stress Ulcers in the Rat. Psychol Rep (1978) 43:591–4. doi: 10.2466/pr0.1978.43.2.591 724904

[88] Achamrah N Nobis S Goichon A Breton J Legrand R do Rego JL. Sex Differences in Response to Activity-Based Anorexia Model in C57Bl/6 Mice. Physiol Behav (2017) 170:1–5. doi: 10.1016/j.physbeh.2016.12.014 27979627

[89] Striegel-Moore RH Rosselli F Perrin N DeBar L Wilson GT May A. Gender Difference in the Prevalence of Eating Disorder Symptoms. Int J Eat Disord (2009) 42:471–4. doi: 10.1002/eat.20625 PMC2696560 19107833

[90] Lutter M Sakata I Osborne-Lawrence S Rovinsky SA Anderson JG Jung S. The Orexigenic Hormone Ghrelin Defends Against Depressive Symptoms of Chronic Stress. Nat Neurosci (2008) 11:752–3. doi: 10.1038/nn.2139 PMC2765052 18552842

[91] Broglio F Arvat E Benso A Gottero C Muccioli G Papotti M. Ghrelin, a Natural GH Secretagogue Produced by the Stomach, Induces Hyperglycemia and Reduces Insulin Secretion in Humans. J Clin Endocrinol Metab (2001) 86:5083. doi: 10.1210/jcem.86.10.8098 11600590

[92] Sun Y Butte NF Garcia JM Smith RG. Characterization of Adult Ghrelin and Ghrelin Receptor Knockout Mice Under Positive and Negative Energy Balance. Endocrinology (2008) 149:843–50. doi: 10.1210/en.2007-0271 PMC2219310 18006636

[93] Zhao T-J Liang G Li RL Xie X Sleeman MW Murphy AJ. Ghrelin O-Acyltransferase (GOAT) is Essential for Growth Hormone-Mediated Survival of Calorie-Restricted Mice. Proc Natl Acad Sci (2010) 107:7467–72. doi: 10.1073/pnas.1002271107 PMC2867684 20231469

[94] Mani BK Zigman JM. Ghrelin as a Survival Hormone. Trends Endocrinol Metab (2017) 28:843–54. doi: 10.1016/j.tem.2017.10.001 PMC5777178 29097101

[95] Miletta MC Iyilikci O Shanabrough M Šestan-Peša M Cammisa A Zeiss CJ. AgRP Neurons Control Compulsive Exercise and Survival in an Activity-Based Anorexia Model. Nat Metab (2020) 2:1204–11. doi: 10.1038/s42255-020-00300-8 33106687

[96] M’Kadmi C Leyris J-P Onfroy L Galés C Saulière A Gagne D. Agonism, Antagonism, and Inverse Agonism Bias at the Ghrelin Receptor Signaling. J Biol Chem (2015) 290:27021–39. doi: 10.1074/jbc.M115.659250 PMC4646384 26363071

[97] Kamegai J Tamura H Shimizu T Ishii S Sugihara H Wakabayashi I. Central Effect of Ghrelin, an Endogenous Growth Hormone Secretagogue, on Hypothalamic Peptide Gene Expression. Endocrinology (2000) 141:4797–800. doi: 10.1210/endo.141.12.7920 11108296

[98] Shintani M Ogawa Y Ebihara K Aizawa-Abe M Miyanaga F Takaya K. Ghrelin, an Endogenous Growth Hormone Secretagogue, Is a Novel Orexigenic Peptide That Antagonizes Leptin Action Through the Activation of Hypothalamic Neuropeptide Y/Y1 Receptor Pathway. Diabetes (2001) 50:227–32. doi: 10.2337/diabetes.50.2.227 11272130

[99] Tschöp M Smiley DL Heiman ML. Ghrelin Induces Adiposity in Rodents. Nature (2000) 407:908–13. doi: 10.1038/35038090 11057670

[100] Zigman JM Nakano Y Coppar R Balthasar N Marcus JN Lee CE. Mice Lacking Ghrelin Receptors Resist the Development of Diet-Induced Obesity. J Clin Invest (2005) 115:3564–72. doi: 10.1172/JCI26002 PMC1297251 16322794

[101] Labarthe A Zizzari P Fiquet O Lebrun N Veldhuis JD Roelfsema F. Effect of GHS-R Deletion on Growth, Pulsatile GH Secretion and Meal Pattern in Male and Female Mice. Neuroendocrinology (2021). doi: 10.1159/000516147 33774644

[102] Verhagen LAW Egecioglu E Luijendijk MCM Hillebrand JJG Adan RAH Dickson SL. Acute and Chronic Suppression of the Central Ghrelin Signaling System Reveals a Role in Food Anticipatory Activity. Eur Neuropsychopharmacol (2011) 21:384–92. doi: 10.1016/j.euroneuro.2010.06.005 20620030

[103] Wortley KE del Rincon J-P Murray JD Garcia K Iida K Thorner MO. Absence of Ghrelin Protects Against Early-Onset Obesity. J Clin Invest (2005) 115:3573–8. doi: 10.1172/JCI26003 PMC1297252 16322795

[104] Carlini VP Monzón ME Varas MM Cragnolini AB Schiöth HB Scimonelli TN. Ghrelin Increases Anxiety-Like Behavior and Memory Retention in Rats. Biochem Biophys Res Commun (2002) 299:739–43. doi: 10.1016/s0006-291x(02)02740-7 12470640

[105] Currie PJ Khelemsky R Rigsbee EM Dono LM Coiro CD Chapman CD. Ghrelin is an Orexigenic Peptide and Elicits Anxiety-Like Behaviors Following Administration Into Discrete Regions of the Hypothalamus. Behav Brain Res (2012) 226:96–105. doi: 10.1016/j.bbr.2011.08.037 21907737 PMC4104180

[106] Hansson C Haage D Taube M Egecioglu E Salomé N Dickson SL. Central Administration of Ghrelin Alters Emotional Responses in Rats: Behavioural, Electrophysiological and Molecular Evidence. Neuroscience (2011) 180:201–11. doi: 10.1016/j.neuroscience.2011.02.002 21303683

[107] Dubois A Gross HA Ebert MH Castell DO. Altered Gastric Emptying and Secretion in Primary Anorexia Nervosa. Gastroenterology (1979) 77:319–23. doi: 10.1016/0016-5085(79)90285-3 376392

[108] Hetterich L Mack I Giel KE Zipfel S Stengel A. An Update on Gastrointestinal Disturbances in Eating Disorders. Mol Cell Endocrinol (2019) 497:110318. doi: 10.1016/j.mce.2018.10.016 30359760

[109] Masuda Y Tanaka T Inomata N Ohnuma N Tanaka S Itoh Z. Ghrelin Stimulates Gastric Acid Secretion and Motility in Rats. Biochem Biophys Res Commun (2000) 276:905–8. doi: 10.1006/bbrc.2000.3568 11027567

[110] Furness JB Pustovit RV Syder AJ Ringuet MT Yoo EJ Fanjul A. Dopamine and Ghrelin Receptor Co-Expression and Interaction in the Spinal Defecation Centers. Neurogastroenterol Motil (2021) 33:e14051. doi: 10.1111/nmo.14051 33264473

[111] Perelló M Zigman JM. The Role of Ghrelin in Reward-Based Eating. Biol Psychiatry (2012) 72:347–53. doi: 10.1016/j.biopsych.2012.02.016 PMC3388148 22458951

[112] Ball KT Combs TA Beyer DN. Opposing Roles for Dopamine D1- and D2-Like Receptors in Discrete Cue-Induced Reinstatement of Food Seeking. Behav Brain Res (2011) 222:390–3. doi: 10.1016/j.bbr.2011.03.064 21497172

[113] Skov LJ Jensen M Christiansen SH Ratner C Woldbye DPD Holst B. Exploring the Behavioral and Metabolic Phenotype Generated by Re-Introduction of the Ghrelin Receptor in the Ventral Tegmental Area. Int J Mol Sci (2017) 18:914. doi: 10.3390/ijms18050914 PMC5454827 28445429

[114] Park S-B King S MacDonald D Wilson A MacKay H Woodside B. Contribution of Growth Hormone Secretagogue Receptor (GHSR) Signaling in the Ventral Tegmental Area (VTA) to the Regulation of Social Motivation in Male Mice. Trans Psychiatry (2021) 11:230. doi: 10.1038/s41398-021-01350-6 PMC8058340 33879778

[115] Bulik CM Beidel DC Duchmann E Weltzin TE Kaye WH. An Analysis of Social Anxiety in Anorexic, Bulimic, Social Phobic, and Control Women. J Psychopathol Behav Assess (1991) 13:199–211. doi: 10.1007/BF00960784

[116] Damian M Louet M Gomes AAS M’Kadmi C Denoyelle S Cantel S. Allosteric Modulation of Ghrelin Receptor Signaling by Lipids. Nat Commun (2021) 12:3938. doi: 10.1038/s41467-021-23756-y 34168117 PMC8225672

[117] Caspar-Bauguil S Montastier E Galinon F Frisch-Benarous D Salvayre R Ritz P. Anorexia Nervosa Patients Display a Deficit in Membrane Long Chain Poly-Unsaturated Fatty Acids. Clin Nutr (2012) 31:386–90. doi: 10.1016/j.clnu.2011.11.015 22385730

[118] Popiołek-Kalisz J Teter M Kozak G Powrózek T Mlak R Sobieszek G. Potential Bioelectrical Impedance Analysis (BIA) Parameters in Prediction Muscle Strength in Women With Anorexia Nervosa. World J Biol Psychiatry (2021) 22. doi: 10.1080/15622975.2020.1774652 32462960

